# EZH2 and BMI1 inversely correlate with prognosis and TP53 mutation in breast cancer

**DOI:** 10.1186/bcr2214

**Published:** 2008-12-19

**Authors:** Alexandra M Pietersen, Hugo M Horlings, Michael Hauptmann, Anita Langerød, Abderrahim Ajouaou, Paulien Cornelissen-Steijger, Lodewijk F Wessels, Jos Jonkers, Marc J van de Vijver, Maarten van Lohuizen

**Affiliations:** 1 Division of Molecular Genetics, The Netherlands Cancer Institute, Plesmanlaan 121, Amsterdam, 1066X, The Netherlands; 2 Current address: Division of Cellular and Molecular Research, National Cancer Centre, 11 Hospital Drive, 169610, Singapore; 3 Division of Experimental Therapy, The Netherlands Cancer Institute, Plesmanlaan 121, Amsterdam, 1066X, The Netherlands; 4 Division of Molecular Biology, The Netherlands Cancer Institute, Plesmanlaan 121, Amsterdam, 1066X, The Netherlands; 5 Department of Genetics, Institute for Cancer Research, Norwegian Radium Hospital, Rikshospitalet University Hospital, Ullernchausèen 70, Motebello 0310, Oslo, Norway; 6 Department of Pathology, Academic Medical Center, Meibergdreef 9, 1105AZ, Amsterdam, The Netherlands

## Abstract

**Introduction:**

PolycombGroup (PcG) proteins maintain gene repression through histone modifications and have been implicated in stem cell regulation and cancer. EZH2 is part of Polycomb Repressive Complex 2 (PRC2) and trimethylates H3K27. This histone mark recruits the BMI1-containing PRC1 that silences the genes marked by PRC2. Based on their role in stem cells, EZH2 and BMI1 have been predicted to contribute to a poor outcome for cancer patients.

**Methods:**

We have analysed the expression of EZH2 and BMI1 in a well-characterised dataset of 295 human breast cancer samples.

**Results:**

Interestingly, although EZH2 overexpression correlates with a poor prognosis in breast cancer, BMI1 overexpression correlates with a good outcome. Although this may reflect transformation of different cell types, we also observed a functional difference. The PcG-target genes INK4A and ARF are not expressed in tumours with high BMI1, but they are expressed in tumours with EZH2 overexpression. ARF expression results in tumour protein P53 (TP53) activation, and we found a significantly higher proportion of TP53 mutations in tumours with high EZH2. This may explain why tumours with high EZH2 respond poorly to therapy, in contrast to tumours with high BMI1.

**Conclusions:**

Overall, our data highlight that whereas EZH2 and BMI1 may function in a 'linear' pathway in normal development, their overexpression has different functional consequences for breast tumourigenesis.

## Introduction

PolycombGroup (PcG) proteins are transcriptional repressors that contribute to the maintenance of cellular identity. Interestingly, several members of the PcG family have been implicated in stem cell regulation and malignancy, including breast cancer [1]. PcG proteins function in distinct multi-protein complexes, which can be roughly distinguished into a silencing initiation complex (Polycomb Repressive Complex 2 (PRC2)) and a complex involved in maintenance of gene silencing (PRC1). The core members of PRC2 are EZH2, SUZ12 and EED. The PRC1 complex, which exhibits a more variable composition, includes BMI1 and RING1b [2]. PRC2-member EZH2 catalyses the histone mark characteristic for PcG-mediated silencing: trimethylation of lysine 27 in histone H3 (H3K27me3) [3]. This mark is required for the recruitment of the PRC1 complex [4,5].

BMI1 is a crucial member of the PRC1 complex and determines the extent of repression of PcG target genes [6-8]. For instance, wildtype BMI1 levels prevent premature expression of the INK4a/Arf locus [5,9]. This region encodes two tumour suppressor genes, p16^INK4a ^and p14^Arf^, that are normally not expressed. When oncogene activation, such as overexpression of c-Myc, occurs a tumour-protective response is triggered in the cell, causing expression of INK4a and ARF. INK4a expression results in activation of the retinoblastoma protein (Rb) that induces cell cycle arrest, whereas ARF expression results in stabilisation of tumour protein P53 (TP53) that can cause either cell cycle arrest or apoptosis [10,11]. However, overexpression of BMI1 can keep the tumour suppressive INK4a/ARF locus silenced, even in the presence of oncogenic signaling, providing an explanation for the collaboration between BMI1 and c-Myc in tumourigenesis [7].

In Bmi1-knockout mice, Ink4a and Arf are expressed at an early age, resulting in premature senescence and progressive loss of adult stem cells [1,12]. Bmi1 was shown to be required for the maintenance of haematopoietic and neural stem cells, through repression of the Ink4a/Arf locus and other PcG target genes [12-14]. We recently showed that loss of Bmi1 reduces stem cell activity in the mammary gland as well, although Bmi1 is also required for the proliferation of more committed cells [6]. The role of Bmi1 in adult stem cells, combined with its frequent overexpression in cancer, has led to the theory that BMI1 might play a role in cancer stem cells (or more accurately, tumour-initiating cells) [15,16]. These cells are thought to be more resistant to therapy [17-19] and a BMI1 overexpression signature has subsequently been correlated with poor prognosis in several tumour types, including breast cancer [15]. However, more recent reports suggest that BMI1 overexpression is associated with a good outcome in breast cancer [1,2].

In contrast, the link between EZH2 overexpression and reduced breast cancer survival is well established. EZH2 overexpression correlates with late stage disease [3,4] and can even be an independent predictor of aggressive breast cancer [5,6]. A causal link between EZH2 overexpression and a malignant phenotype was observed in human papillomavirus-transformed human mammary epithelial cells where EZH2 overexpression conferred anchorage-independent growth and invasive properties to these cells [3]. In mouse development, deletion of EZH2 is embryonic lethal. EZH2 was shown to be required for embryonic stem cells [7], and although less is known about EZH2 function in adult stem cells, it does play a role in haematopoietic stem cells [8]. In the mammary gland, EZH2 expression is coupled with proliferation, but in tumours EZH2 expression is also found in resting cells [4].

In light of the potential role of PcG proteins in cancer stem cells [9,10], we investigated the controversial role of BMI1 in breast cancer and compared it with EZH2 to obtain more of an insight into polycomb function in tumourigenesis. We found that in contrast to EZH2, BMI1 overexpression correlates with a good prognosis. Moreover, we found inverse correlations for BMI1 and EZH2 expression with several clinical characteristics, as well as different tumour subtypes. Finally, we identified a possible mechanism to explain why BMI1 overexpression may contribute to a better outcome than EZH2 overexpression in breast cancer.

## Materials and methods

### Tumour samples and patient characteristics

We have previously published the gene expression profiles of tumours from 295 stage 1 and stage 2 breast cancer patients treated at the Netherlands Cancer Institute between 1984 and 1995 [11]. Data on overall survival was available for all patients [11] and clinical information was last updated on 1 January 2005. The median follow up is 10.2 years for all patients and 12 years for patients who are alive. One hundred and sixty-five patients received local therapy, 20 received tamoxifen only, 20 received tamoxifen plus chemotherapy and 90 received chemotherapy.

### Classification of tumour samples according to gene expression

Expression data was obtained from 25,000 oligo arrays comparing complementary RNA (cRNA) from individual tumour samples with a pool containing equal amounts of cRNA from all patients [12,13]. For categorisation into high and low expression groups for EZH2 [GenBank:NM004456], BMI1 [GenBank:NM005180], EED [GenBank:AF099032] and RING1B [RNF2, GenBank:NM007212], we chose a log2 ratio of 0 as the cut-off point, which resulted in 40%, 38%, 44% and 52% of patients having high levels of expression, respectively.

Classification of intrinsic molecular subtypes was carried out according to new guidelines [14]. Patients were furthermore categorised based on the 70-gene signature [12], death-from-cancer signature [15] and Genomic Health Score [16].

### Statistical analysis

Time-to-event data were illustrated by Kaplan-Meier plots and groups were compared using the Cox-Mantel log-rank test. Overall survival was defined as the time from age at diagnosis to age at death or end of follow up, whichever occurred first. Cox proportional hazards regression analyses were used to calculate uni- and multivariate hazard ratios (HR) and their 95% confidence intervals (CI). Multivariate HRs were adjusted for tumour grade, nodal status and tumour diameter. Associations between categorical variables were evaluated by Pearson's chi-squared test or by logistic regression. Statistical significance was assessed at the two-sided 5% level. All statistical analysis was done using SAS 9.1 (SAS Institute Inc., Cary, NC, USA).

### Immunohistochemistry

For all breast carcinomas, BMI1, EZH2, oestrogen receptor (ER), human epidermal growth factor receptor (HER) 2 and TP53 status were assessed by immunohistochemistry on tissue microarrays using a manual tissue arrayer (Beecher Instruments, Inc, Sun Prairie, WI, USA). From each individual paraffin-embedded tumour, 600 μm core tissue biopsies were taken and arrayed in triplicate in a new paraffin block. Serial sections of 3 μm were cut from the tissue microarray blocks, deparaffinised in xylene and hydrated in a graded series of alcohol. After antigen retrieval in citrate buffer, staining was performed using the Lab Vision Immunohistochemical Autostainer (Lab Vision Products

Thermo Fisher Scientific, Fremont, CA, USA) with primary antibodies against ER-alpha (1D5+6F11, dilution 1:50, Neomarkers (Lab Vision Products

Thermo Fisher Scientific, Fremont, CA, USA)), HER2 (3B5, dilution 1:3000), TP53 (DO-7, dilution 1:6000), EZH2 (AE25, dilution 1:10, gift from Kristian Helin) and BMI1 (F6, dilution 1:400, Millipore). ER-staining was scored as positive when more than 10% of tumour cells showed staining, and TP53 staining was considered positive when more than 50% of tumour cells showed staining. A tumour was considered HER2 positive with a score of 3+ and negative with a score of 0, according to common pathological guidelines. Tumours with a score of 2+ were evaluated by chromogenic in situ hybridisation (CISH) and scored positive when HER2 gene amplification was found.

### TP53 mutation analysis

When RNA was isolated from tumour tissue using TRIzol reagent (Invitrogen, Breda, Netherlands), the DNA fraction was used for TP53 mutation analysis. TP53 mutation detection in tumour DNA was performed by sequencing exon 2 to 11 using the 3730 DNA analyser (Applied Biosystems Inc, Foster City, CA, USA). All fragments were sequenced in both directions and the primers used were linked to universal M13 sequences. PCR products were purified with MultiScreen PCRμ96 Plate (Millipore) on epMotion™5075 (Eppendorf, Westbury, NY, USA). In a 20 μl PCR reaction, 50 ng DNA was amplified using HotStarTaq DNA Polymerase (Qiagen, Valencia, CA, USA) and touchdown PCR with annealing temperatures from 68°C to 56°C. BigDye Terminator reaction mix v1.1 was used in the sequencing reacion, and the products were purified with Sephadex G-50 Superfine. SeqScape software v2.5 (Applied Biosystems Inc, Foster City, CA, USA) was used for alignment to reference sequence and the scoring were carried out by two experienced operators independently. GeneBank accession number [Genbank:NM000546] was used as a reference sequence.

## Results

### Inverse correlation of PRC1 and PRC2 members with survival

We evaluated the association between BMI1 or EZH2 expression and overall survival among 295 breast cancer patients [11,12]. For each gene, we divided patients into a high and low expression group as determined by microarray analysis.

**Figure 1:**
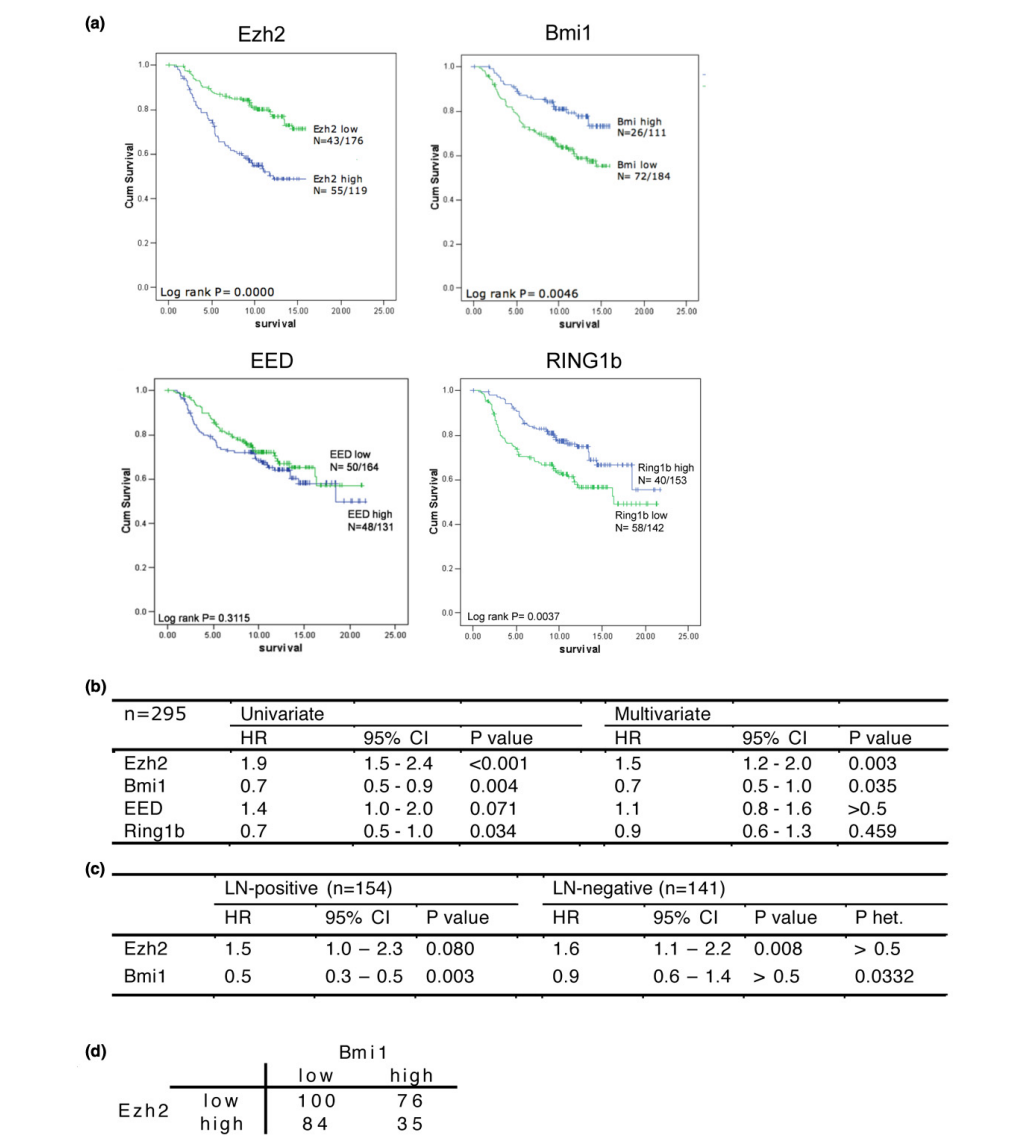
**EZH2 and BMI1 are inversely correlated**. (a). Univariate survival analyses by Kaplan-Meier plots and log rank tests. Patients were categorised by high or low expression of the different Polycomb group (PcG) members based on microarray data. (b) Relative risk of death evaluated by Cox regression modelling and continous PcG mRNA levels. Multivariate analysis is adjusted for tumour size, lymph node (LN) status and grade. (c) Relative risk of death based on continous EZH2 and BMI1 levels in LN-positive and LN-negative patients. P het = p value indicating heterogeneity of BMI1 levels across LN-positive and LN-negative patients. (d) Two-by-two table for categorical EZH2 and BMI1 levels. Odds ratio (OR) = 0.55 (95% confidence interval (CI) = 0.33 to 0.90) for high EZH2 among patients with high BMI1 compared with low BMI1 (chi-squared: p = 0.017).

Univariate survival analysis corroborated previous data [3,5] that patients with high EZH2 expression have a shorter overall survival than patients with low EZH2 (Figure 1a). In contrast, patients with high expression of BMI1 showed increased overall survival. Two other PcG members that showed an effect on overall survival are EED and RING1B (RNF2). Interestingly, high expression of EED, which like EZH2 is a PRC2 member, is also associated with a shorter survival time whereas high expression of RING1B, the binding partner for BMI1, is correlated with a favourable disease outcome. Similar associations were found when the mRNA expression was regarded as a continuous variable (Figure 1b). However, after adjustment for clinical characteristics, only EZH2 and BMI1 remain significantly associated with survival time, albeit in opposite directions. The HR for EZH2 is not affected by the lymph node status of the patients, but the 'protective' effect of BMI1 expression is limited to lymph node-positive patients (Figure 1c).

The differential association with survival suggests that EZH2 and BMI1 are not overexpressed in the same tumours. As illustrated in Figure 1d, there is indeed a negative correlation between EZH2 and BMI1 expression. Although there does not seem to be absolute incompatibility between high levels of BMI1 and EZH2, tumours with high BMI1 have only about half the chance to have high EZH2 compared with tumours with low BMI1 (odds ratio (OR) = 0.55, p = 0.0166). Of note, tumours with both high EZH2 and BMI1 expression have a HR of 0.6 (95% CI = 0.3 to 1.2) compared with tumours that have high EZH2 and low BMI1, suggesting that the favourable effect of BMI1 overexpression is dominant over the adverse effects of EZH2.

### BMI1 correlates with favourable tumour characteristics

**Figure 2:**
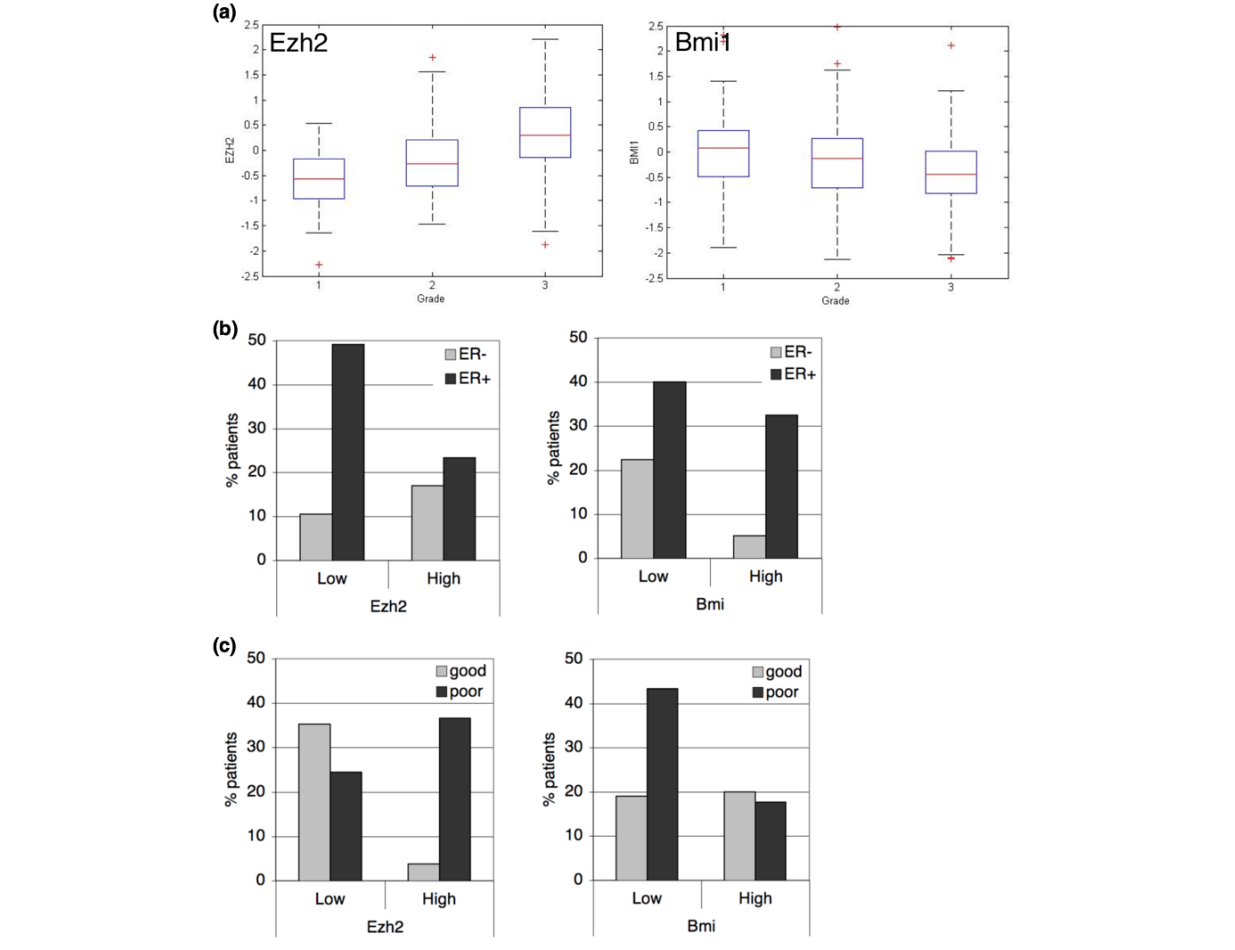
**EZH2 and BMI1 inversely correlate with variables associated with survival**. (a) EZH2 and BMI1 distribution differ by grade. Mean EZH2 mRNA levels are -0.62 (n = 75), -0.21 (n = 101) and 0.37 (n = 119) for grades 1, 2 and 3, respectively (p < 0.0001 Kruskal-Wallis test). Mean BMI1 mRNA levels are 0.0067, -0.15 and -0.41 for grades 1, 2 and 3, respectively (p = 0.0001 Kruskal-Wallis test). (b) EZH2 and BMI1 distribution differ by oestrogen receptor (ER) status. Tumours are regarded ER-positive when more than 10% of the tumour cells stain positive for ER immunohistochemistry and ER-negative otherwise. Mean EZH2 levels are 0.34 for ER-negative (n = 81) tumours and -0.27 for ER-positive (n = 214) tumours (p < 0.0001 Wilcoxon test). Mean BMI1 levels are -0.56 for ER-negative and -0.08 for ER-positive tumours (p < 0.0001 Wilcoxon test). (c) EZH2 and BMI1 distribution differ by 70-gene profile. Mean EZH2 levels are -0.58 for tumours with a good prognosis signature (n = 115) and 0.22 for tumours with a poor prognosis signature (n = 180) (p < 0.0001 Wilcoxon test). Mean BMI1 levels are -0.005 for good prognosis tumours and -0.35 for poor prognosis tumours (p < 0.0001 Wilcoxon test).

To obtain further insight into the differential associations of BMI1 and EZH2 with overall survival, we analysed the mRNA expression levels of these PcG genes in tumours ranked according to grade (Figure 2a). Grade 3 tumours, which have a worse prognosis than grade 1 and 2 tumours, expressed significantly more EZH2 and less BMI1 than tumours of lower grade (Kruskal-Wallis test, p = 0.001). Although we observed an effect of BMI1 in lymph node-positive patients only, there is no significant variation in the expression levels of BMI1 or EZH2 according to lymph node status or age at diagnosis (data not shown). Next, we tested whether PcGs are associated with ER status. High BMI1 expression comes with a significantly higher frequency of ER-positive tumours (Wilcoxon test p < 0.001), which seems to be mainly due to a lack of BMI1 expression in ER-negative tumours (Figure 2b). In contrast, high EZH2 expression is negatively correlated with ER expression (Wilcoxon test p < 0.001).

EZH2 is one of a set of 70 genes whose expression predicts a poor outcome in breast cancer [12], so it is not surprising that most patients with high EZH2 exhibit this poor prognosis signature. Conversely, patients with a poor 70-gene profile are overrepresented in the category with low BMI1 expression, consistent with our observations that low BMI1 expression is associated with decreased overall survival (Figure 2c).

EZH2 has been linked to proliferation in several tumour types [5,6]. We also find a positive correlation between EZH2 and proliferation (Pearson correlation 0.61) in our dataset when using the five genes from the Genomic Health Score [16] to reflect the proliferation status of the tumours. Conversely, BMI1 expression is negatively correlated (-0.27) with tumour cell proliferation.

An 11-gene signature, based on a mouse model of metastatic prostate cancer and on BMI1-deficient versus BMI1-proficient neurospheres, has been suggested to reflect BMI1-regulated 'stem cell-ness' pathways and to predict a poor outcome in breast cancer [15]. In our analysis, however, this 11-gene signature showed a negative correlation with BMI1 expression (Pearson correlation coefficient -0.24) and a positive correlation with EZH2 expression (correlation coefficient 0.47), suggesting that this signature does not reflect BMI1 function.

Overall, tumours with high BMI1 expression display characteristics of less malignant tumours and are correlated with increased survival.

### High BMI1 expression is restricted to luminal breast cancer subtypes

Breast cancer is a heterogeneous disease and tumours are classified into distinct subgroups based on morphology or gene expression profiles. The molecular subtypes identified by Perou and colleagues [17] and Sørlie and colleagues [18] showed a striking difference in survival. Furthermore, it has been postulated that the differences between breast cancer subgroups may be due to the fact that they derive from different cells of origin [19,20]. For instance, the basal subtype expression profile harbors characteristics of undifferentiated cells, whereas the luminal A subtype expresses genes indicative of luminal epithelial cells such as mucin and ER. It is possible that these distinct tumours arise from the oncogenic transformation of a mammary stem cell or a more committed luminal progenitor, respectively. This idea fits with a recent publication that demonstrated that the cell type, or epigenetic make-up, determines for a large part the phenotype and malignancy of the resulting tumour [21].

**Figure 3:**
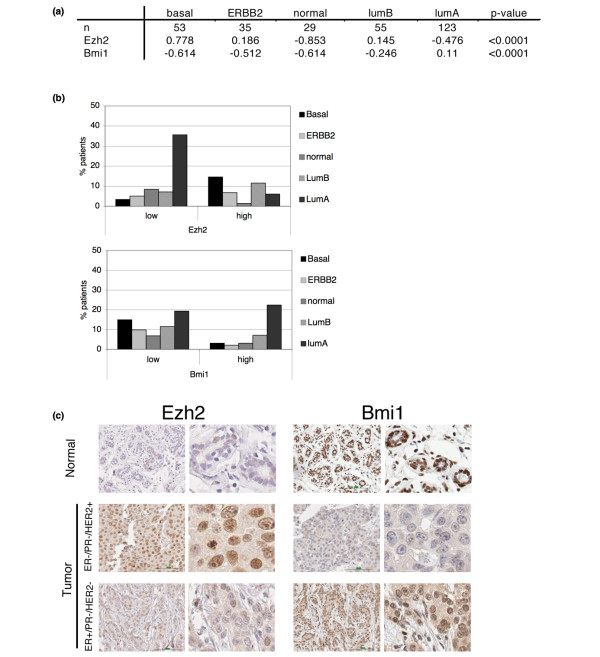
**Correlation of EZH2 and BMI1 expression with molecular subtypes**. (a) Mean mRNA levels of EZH2 and BMI1 in the different subgroups. The distribution of both EZH2 and BMI1 over the subgroups differ significantly, Kruskall-Wallis test. (b) Distribution of subgroups over categorised EZH2 and BMI1 levels. (c) Immunohistochemistry on EZH2 and BMI1 in normal breast tissue, an ErbB2-type and a luminal A-type tumour. Pictures were taken at 40× magnification (left panels) and a zoomed-in section is shown on the right of each image.

Therefore, we explored whether EZH2 or BMI1 expression was associated with a specific molecular subtype. When ranked according to prognosis, the basal subtype has the worst prognosis, followed by the Erb-B2+, normal-like and luminal B tumours. The luminal A subtype has by far the best prognosis [14,18]. We found a substantial heterogeneity of EZH2 and BMI1 levels across the subtypes (p < 0.0001, Figure 3a). Analysis of the distribution of the distinct molecular subtypes in patients with either high or low BMI1 expression demonstrates that whereas all subtypes are represented in the BMI1-low group, the BMI1-high group consists mainly of the luminal subtypes, particularly luminal A (Figure 3b). This is consistent with the association of BMI1 with ER-positive tumours as luminal A tumours are usually ER-positive and luminal B tumours generally show a lower ER-expression than luminal A [18]. For EZH2, we find an under-representation of the normal-like subtype in tumours with high EZH2-expression, and an overrepresentation of luminal A type tumours in the group with low EZH2 expression (Figure 3b).

The heterogeneity of the different breast cancer subtypes with respect to overall survival, and the possibility that tumours of different subtypes may derive from different cells of origin, suggest that the expression levels of EZH2 and BMI1 in breast tumours reflect a pre-existing expression pattern in different cell types within the mammary gland. Therefore, we stained normal mammary gland tissue as well as breast tumour material for both BMI1 and EZH2 protein expression. BMI1 was expressed in virtually all cells of the mammary gland, but particularly high in luminal cells (Figure 3c), similar to what we observed in mouse mammary tissue [22]. EZH2 expression seems to be low in general (Figure 3c) and is possibly restricted to proliferating cells only, as reported previously [4]. Alternatively, EZH2 expression may be restricted to a less abundant cell population such as, for instance, stem cells.

We have performed immunohistochemistry (IHC) on a tissue microarray from the same 295 patients that were used for the microarray-based analyses. However, correlation of the categorical BMI1 IHC groups with the continuous mRNA variable is low, although the correlation for EZH2 is somewhat better. Careful analysis by an experienced pathologist (MJV) indicated that the BMI1 IHC signal is high in most cells and there is a limited range of BMI1 expression, precluding a reliable categorisation of the samples based on IHC. A higher variation of EZH2 staining intensity between samples partially overcomes this problem. Nevertheless, we found that both BMI1 and EZH2 epitopes are particularly sensitive to the age of the tissue, as well as the time between sectioning and staining. Consequently, even though the staining pattern within one patient sample is informative, we have based our comparison of the different tumour samples on the more quantitative mRNA expression data.

### Low frequency of TP53 mutations in tumours with high BMI1 expression

**Figure 4:**
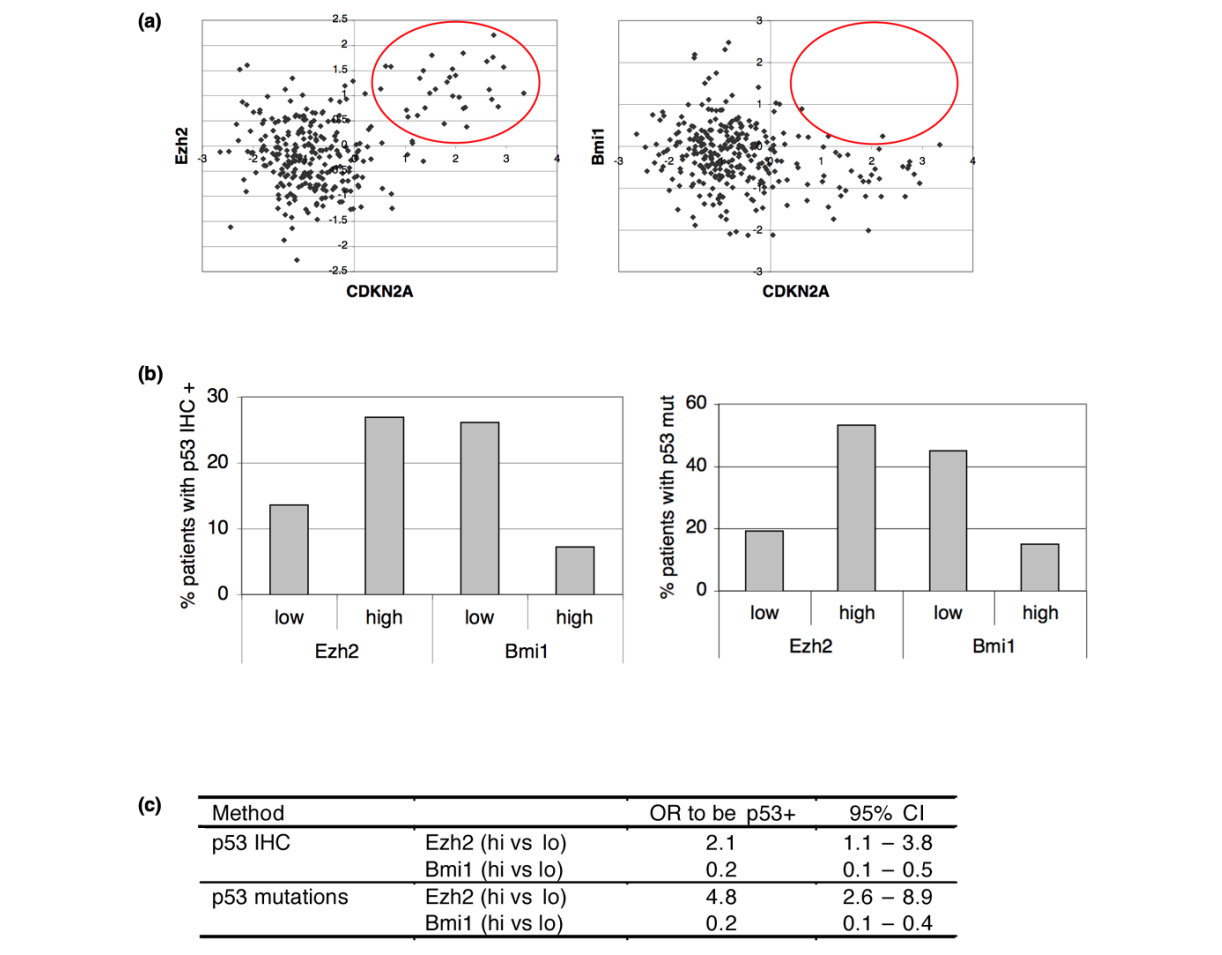
**Differential effect of BMI1 and EZH2 on INK4a/ARF locus and association with TP53 mutations**. (a) Dotplot of INK4a/ARF (CDKN2A) expression versus BMI1 and EZH2 expression. INK4a/ARF expression is not found in tumours with high BMI1 mRNA, whereas it does occur in tumours with high EZH2 (red circle). (b) Distribution of patients with positive TP53 staining (mutated TP53) differs by categorised EZH2 and BMI1 levels. TP53 immunohistochemistry (IHC) data was obtained from 273 patients and the TP53 sequence was analysed in 204 tumours. The distribution of mutant TP53 shows a similar pattern (TP53 mutation). (c) Logistic regression analysis of TP53 IHC and sequence status on EZH2 and BMI1 levels. CI = confidence interval, OR = odds ratio.

Although the inverse correlation of EZH2 and BMI1 with survival may simply reflect the transformation of different cell types, this differential association may also be explained by a functional difference between both Polycomb genes. The INK4a/ARF locus is a known target of polycomb repression, so we analysed expression of this locus in relation to BMI1 and EZH2. As expected, we did not find high INK4a/ARF expression in tumours with high BMI1 mRNA expression (Figure 4a). However, to our surprise, tumours with high EZH2 expression did have high INK4a/ARF expression in 33% of the cases. This suggests that while overexpression of BMI1 is capable of blocking oncogene-induced INK4a/ARF expression, this is not the case for EZH2 overexpression. A consequence of ARF induction is stabilisation of TP53 [23], resulting in apoptosis or cell cycle arrest. Thus, cells expressing high EZH2 levels would come under selection for mutations in TP53 once they progress to malignancy [24]. In contrast, there would be no selection pressure towards mutant TP53 in tumours in which high BMI1 levels block INK4a/ARF expression induced by oncogenic stress.

To test this hypothesis, we analysed TP53 status in our patient data set. Positive TP53 staining as detected by IHC is frequently used as an indicator of mutated TP53 [25]. However, because not all mutations result in increased expression, we also used TP53-mutation status based on sequencing (for 204 of the 295 patients). Consistent with the increased ARF expression, we observed more tumours with a strong TP53 staining or a mutant TP53 sequence in the EZH2-high group (Figure 4b). In contrast, tumours with high BMI1 are five times less likely to have mutated TP53 (Figure 4c). To further substantiate this observation, we made use of a gene expression signature that was found to reflect TP53 status in breast cancer [26]. Again, we found significantly more tumours with a mutant TP53 signature when EZH2 expression is high [see Additional data file 1].

## Discussion

BMI1 has been proposed to play a role in cancer stem cells or tumour-initiating cells [10,27] because of its function in normal stem cells and its role as an oncogene. It has been postulated that cancer stem cells are particularly resistant to therapy [28], so high levels of BMI1 would be expected to be associated with a poor prognosis. In several tumour types, such as leukaemia and hepatocellular carcinoma, BMI1 overexpression is indeed correlated with reduced survival [29,30]. However, our data demonstrated that in breast cancer this is not the case. In fact increased BMI1 expression is associated with a good prognosis. Two recent reports also documented the association of BMI1 expression with survival after breast cancer. In both datasets BMI1 expression is associated with ER-positive disease, and in one set BMI1 is an independent predictor of good prognosis [1,2]. These studies used IHC and show a reasonable correlation with mRNA expression, indicating that although the range of BMI1 protein detection may be limited, both mRNA and protein levels give a similar result regarding an association of BMI1 with a positive outcome.

We further show that high BMI1 expression is limited to the luminal breast cancer subtypes, and is inversely correlated with PcG members from the silencing initiation complex, PRC2. There could be several, not mutually exclusive, reasons for the differential association of EZH2 or BMI1 overexpression with overall survival. First, high BMI1 or EZH2 levels may reflect a different cell of origin for the respective tumours. Ince and colleagues recently demonstrated that the introduction of identical transforming mutations in different mammary epithelial cell types resulted in strikingly different oncogenic potential of these cells [21]. This implies that the pre-existing expression patterns or differentiation state of a cell determines how malignant that cell can become. Because we found different expression patterns for EZH2 and BMI1 in the normal mammary gland, it is possible that the expression levels we detected in the tumours are a reflection of BMI1 and EZH2 expression in the cell of origin. The fact that EZH2 is expressed in a small subset of cells in the mammary gland, is found overexpressed in the undifferentiated basal subtype and is associated with a poor prognosis suggests that EZH2 might play a role in mammary stem cells. Nevertheless, EZH2 expression is not limited to a particular subtype and is also linked to the proliferative state of cells: therefore, a possible link between EZH2 and mammary stem cells remains to be demonstrated. Importantly, we find little evidence for a role of BMI1 in the transformation of mammary stem cells. Both in the human and the mouse mammary gland, BMI1/Bmi1 is expressed in all cells, but is particularly high in luminal cells. In the mouse, we observed that loss of Bmi1 reduces mammary stem cell activity, but Bmi1 is also required for the proliferation of more committed cells [22]. In addition, we found that high BMI1 levels are mainly restricted to ER-positive luminal A type tumours. Furthermore, Shipitsin and colleagues did not find an increase in BMI1 in the tumour-intiating population [31]. Therefore, a role for BMI1 in the transformation of luminal progenitors or even more committed luminal epithelial cells is more likely. The differentiation state of these cells may contribute to a less aggressive phenotype after transformation.

Although BMI1 expression does not seem to be restricted to ER-positive cells in normal mammary gland tissue, we found a strong correlation between BMI1 overexpression and positive ER status in the human breast cancer samples. It is possible that ER-positive cells derive a selective advantage from BMI1 overexpression. In the normal mammary gland, ER-positive cells are growth arrested. They sense the growth signal from oestrogen levels, but pass this on via paracrine factors that induce growth in neighbouring ER-negative cells [32,33]. This could be viewed as a tumour-protective strategy and the growth arrest is indeed abrogated in ER-positive tumours, which have become dependent on oestrogen for their proliferation [34,35]. A recent study showed that whereas normal ER-positive cells are rapidly lost in cell culture because of their growth arrest, BMI1 overexpression allows proliferation of cells expressing the ER [36]. It will be interesting to determine if this is due to the repression of the INK4a/ARF locus or if other BMI1-target genes are involved. The strong association of BMI1 overexpression and luminal A type tumours may therefore suggest that BMI1 is selectively oncogenic in ER-positive cells, where it can over-rule their arrested state.

In contrast to BMI1, we found that EZH2 overexpression is negatively correlated with ER expression. In two cell culture systems, EZH2 overexpression was shown to repress and activate ER signaling, respectively [37,38]. However, most ER-positive tumours have low EZH2 and the reported corepressor for EZH2 is downregulated in high-grade tumours, where EZH2 expression is high. Hence, it seems unlikely that involvement of EZH2 in ER-signaling contributes greatly to an oncogenic role of EZH2 in breast cancer.

EZH2 may, however, be involved in oncogenic transformation by attenuating DNA repair, thereby increasing genomic instability [39]. Whereas this could contribute to tumourigenesis by increasing the mutation rate, it was also shown to make cells in culture more sensitive to radiation or etoposide [39]. However, EZH2 was introduced into cell lines with functional TP53, which could explain the increased sensitivity to therapy. Importantly, our data show that primary tumours with EZH2 overexpression frequently overexpress INK4a/ARF and harbor TP53 mutations. Even though ectopic EZH2 may sensitise cells with wildtype TP53 to treatment, our data suggest that during tumourigenesis EZH2 overexpression coincides with TP53 activation, either through genomic instability or ARF induction. Therefore the resulting tumours would derive from clones that have accrued TP53 mutations, rendering them more resistant to therapy, as indicated by the link of EZH2 with poor overall survival.

**Figure 5:**
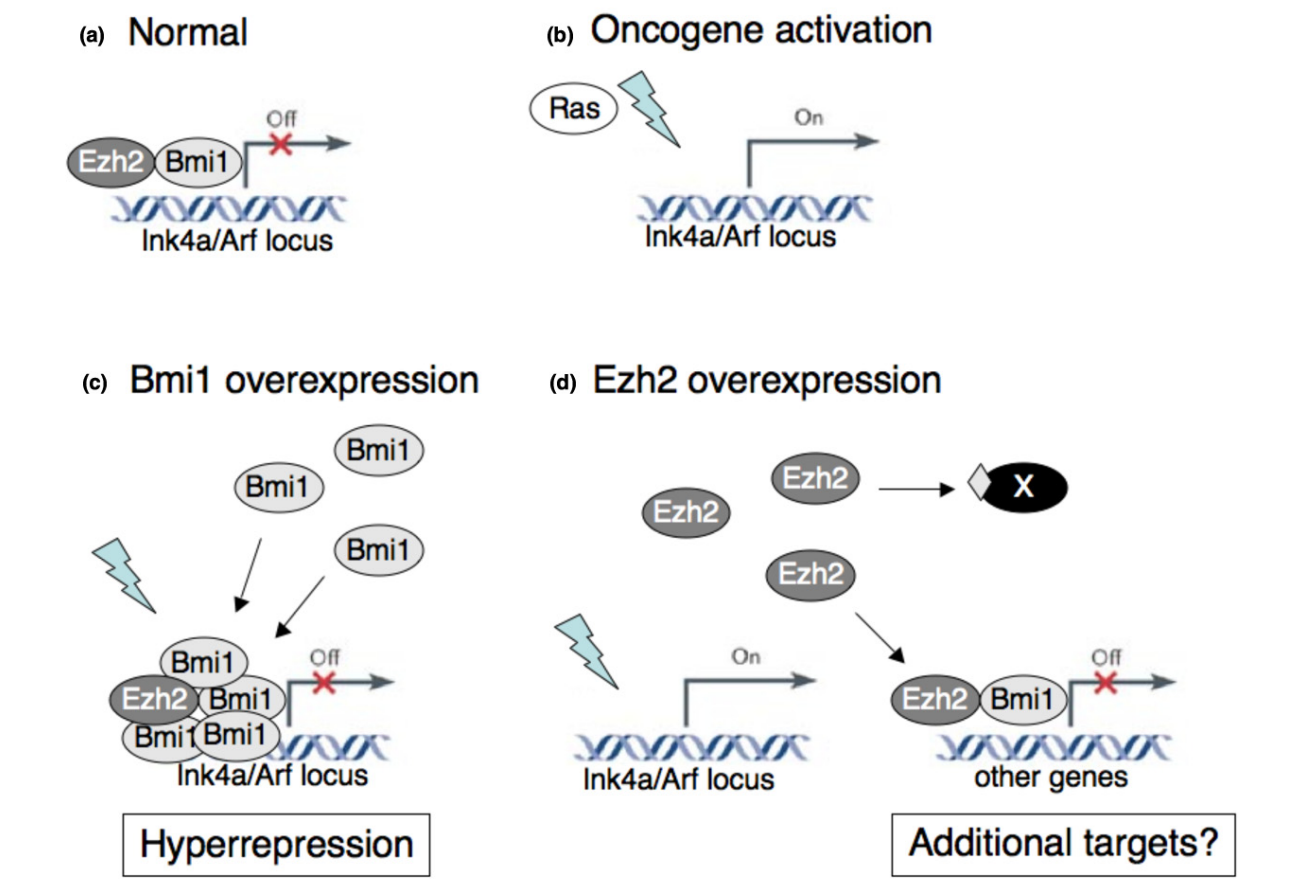
**Model for the different oncogenic roles of EZH2 and BMI1**. (a) In normal development, genes such as the INK4a/ARF locus are bound by EZH2, marked by H3K27me3 and bound by BMI1. (b) On oncogene activation (indicated by lightning symbol) such as constitutive Ras or Myc expression, the INK4a/ARF locus becomes expressed as part of a tumour suppressive response. (c) Increased BMI1 expression results in more BMI1 protein binding to known Polycomb group (PcG)-target genes. This prevents activation of these genes even when signals are present that would normally activate these genes. (d) Increased EZH2 expression does not prevent INK4a/ARF activation (although this locus may still be inactivated by other mechanisms). In contrast, the role of EZH2 in tumourigenesis may be due to silencing of genes not normally targeted by PcG or due to methylation of non-histone proteins (indicated by diamond on protein 'X').

Apparently, EZH2 overexpression is not capable of repressing the INK4a/ARF locus in the presence of oncogenic signaling, unlike BMI1 overexpression. Indeed, in human fibroblasts ectopic EZH2 is not recruited to the INK4a/ARF locus, in contrast to ectopic BMI1 [40]. BMI1 levels determine the extent of repression of its target genes [41], so we propose the following model with regard to the differential roles of BMI1 and EZH2 in breast tumourigenesis (Figure 5). Overexpression of BMI1 results in increased silencing of PcG target genes such as the INK4a/ARF locus. Whereas wildtype levels of BMI1 would not be sufficient to prevent activation of INK4a/ARF by, for instance, oncogenic stress, overexpression of BMI1 can block this activation and thus prevent TP53 activation. In that case, there is no selection pressure towards mutant TP53 in BMI1 overexpressing cells. Importantly, activation of TP53 by DNA damage occurs via ATM/ATR signaling and not via ARF [42,43], which implies that treatment of BMI1-expressing tumours with DNA damaging drugs should activate the TP53 pathway to induce apoptosis. It has been shown that TP53 status can indeed predict response to therapy in breast cancer [44]. Interestingly, the protective effect of BMI1 is not observed in lymph node-negative patients, who undergo resection and local treatment only (Figure 1c). In contrast, BMI1 overexpression associates with a good prognosis for lymph node-positive patients, who receive chemotherapy, hormone therapy or both. Finally, the observation that BMI1 overexpression still confers a protective effect in tumours with high EZH2 supports this model in which BMI1 overexpression prevents TP53 mutations and thus results in a better response to therapy.

For EZH2 on the other hand, our data imply that overexpression of this PcG member can not prevent the activation of the INK4a/ARF locus. We suggest that the selection for TP53 mutations contributes to the poor outcome observed in tumours with high EZH2, but not BMI1, expression.

Possibly, the main molecular function of EZH2 is in 'tagging' which genes need to be silenced rather than silencing the genes itself. Arguably, the INK4a/ARF locus is already saturated by H3K27me3 and increased EZH2 levels do not affect this, whereas an increase in BMI1 levels results in more BMI1 being present at the locus [40]. By nature of its catalytic activity, overexpression of EZH2 could result in the silencing of genes that are normally not PcG targets, as also suggested by Tan and colleagues [45]. This group identified a set of tumour-specific PcG target genes and validated this in primary human breast tumour samples. We could not find a correlation between reduced expression of these genes and high EZH2 expression in our data set (data not shown). It is conceivable that EZH2 is not specifically recruited to new targets, but silences genes at random. Those genes whose silencing contributes to an oncogenic phenotype would subsequently be selected during tumourigenesis. Depending on accompanying mutations, different genes are likely to be selected for silencing in different tumours, making it difficult to detect a specific epigenetic profile for EZH2 overexpression. Nevertheless, it is likely that EZH2 has an INK4a/ARF-independent function in cancer, because in mammary epithelial cells in which Rb and TP53 had been inactivated, ectopic expression of EZH2 contributed to anchorage-independent growth and invasion [3]. Interestingly, EZH2 was found to methylate cytoplasmic proteins involved in Akt-signaling [46], suggesting that the oncogenic role of EZH2 in breast cancer could also be unrelated to its role in epigenetic regulation.

## Conclusion

Overall, our data indicate that important functional differences exist between different PcG proteins. Specifically, although under normal circumstances EZH2 and BMI1 work in a 'linear' pathway, that is, BMI1 recruitment to a target gene depending on the H3K27me3 mark set by EZH2, the overexpression of these genes seems to have very different consequences with regard to, for instance, INK4a/ARF expression and TP53 mutations. Future studies on the causal involvement of PcG proteins in stem cell regulation and tumourigenesis should take into account the molecular function of individual polycomb members as well as potential differences in cells of origin.

## Abbreviations

ARF/Arf: CDKN2A/cdkn2a (cyclin-dependent kinase inhibitor 2A), Alternative Reading Frame, a.k.a. p14/p19; ATM/ATR pathway: ataxia telangiectasia- and Rad 3 related pathway; BMI1/Bmi1: B-cell-specific Moloney murine leukemia virus integration site 1; CDKN2A/Cdkn2a: cyclin-dependent kinase inhibitor 2A; CI: confidence interval; CISH: chromogenic in situ hybridisation; cRNA: complementary RNA; c-Myc: myelocytomatosis oncogene; EED/Eed: embryonic ectoderm development; ER: oestrogen receptor; EZH2/EZH2: enhancer of zeste homologue 2; H3K27: lysine 27 in histone H3; H3K27me3: trimethylation of lysine 27 in histon H3; HER2: Human Epidermal growth factor Receptor 2; HR: Hazard Ratio; IHC: immunohistochemistry; INK4a/Ink4a: CDKN2A/cdkn2a (cyclin-dependent kinase inhibitor 2A), a.k.a. p16, RING1B/Ring1b = RNF2 = ring finger protein 2; OR: odds ratio; PcG: polycomb group; PRC: polycomb repressive complex; Rb: retinoblastoma; SUZ12/Suz12: suppressor of zeste 12 homolog; TP53/Tp53: tumour protein 53.

## Competing interests

The authors declare that they have no competing interests.

## Authors' contributions

AMP and HMH contributed equally to this study. AMP, HMH, MJV, JJ and MvL conceived the study, and participated in its design and coordination. AA and HMH performed immunoassays. PCS performed qPCR validations and AL carried out the TP53 mutation analysis. MH, LFW, AMP and HMH conducted the statistical analysis. AMP and HMH drafted the manuscript. All authors read and approved the final manuscript.

## Supplementary Material

Additional file 1 PDF file containing a figure that shows that Miller's TP53 signature also identifies more TP53 mutations in high EZH2 breast tumours. Click here for file
