# Supplementary material for: EZH2 and BMI1 inversely correlate with prognosis and TP53 mutation in breast cancer
Source: Breast Cancer Res. 2008 Dec 19;10(6):R109. doi: 10.1186/bcr2214 (PMC2656906; doi:10.1186/bcr2214)
Supplement: Additional file 1 — PDF file containing a figure that shows that Miller's TP53 signature also identifies more TP53 mutations in high EZH2 breast tumours. [file bcr2214-S1.pdf]

Figure S1. Miller's TP53 signature also identifies more TP53 mutations in high EZH2 tumors
There is a good correlation between positive TP53 staining by IHC and TP53 expression signature predicting functional inactivation of TP53 (Miller et al PNAS 2005). Patients with high EZH2 have a 3.8-fold chance to have inactive TP53 according to the signature compared to patients with low Ezh2 ($p< 0.0001$). Patients with high BMI1 have a 70% reduced chance to have inactive TP53 according to the signature compared to patients with low BMI1 ($p<0.0001$).

	Miller's p53 signature		
	OR	95% CI	P value
p53 IHC	4.8	2.6-8.8	<0.0001
Ezh2	3.8	2.2-6.4	<0.0001
Bmi1	0.3	0.2-0.5	<0.0001
